# Diiodido[methyl 2-(quinolin-8-yl­oxy)­acetate-κ*N*]mercury(II)

**DOI:** 10.1107/S1600536812031017

**Published:** 2012-07-14

**Authors:** Yu-Hong Wang, Qin Zhong, Rui-Feng Song

**Affiliations:** a School of Chemistry and Bioengineering, Suzhou University of Science and Technology, Suzhou 215009, People’s Republic of China

## Abstract

In the title mononuclear complex, [HgI_2_(C_12_H_11_NO_3_)], the Hg^II^ ion has a distorted trigonal–planar coordination sphere defined by two I^−^ anions and the N atom of a methyl 2-(quinolin-8-yl­oxy)acetate ligand. In the crystal, face-to-face π–π stacking inter­actions, with a centroid–centroid distance of 3.563 (9) Å, are observed.

## Related literature

For derivatives of quinoline, see: Cheng *et al.* (2007 ▶); Ghedini *et al.* (2002 ▶); Inomata *et al.* (1999 ▶); Jotterand *et al.* (2001 ▶). For transition metal coordination compounds of 8-quinolinyloxy­acetic acid, see: Cheng *et al.* (2007 ▶); Song *et al.* (2004 ▶); Wang *et al.* (2005 ▶, 2008 ▶).
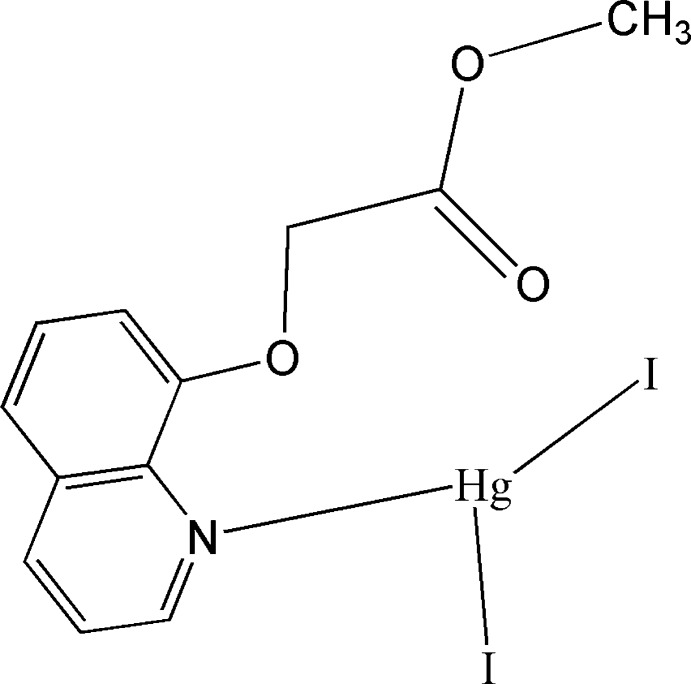


## Experimental

### 

#### Crystal data

[HgI_2_(C_12_H_11_NO_3_)]
*M* *_r_* = 671.61
Triclinic, 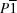

*a* = 7.5889 (5) Å
*b* = 10.3670 (7) Å
*c* = 11.4241 (11) Å
α = 72.203 (18)°
β = 74.40 (2)°
γ = 68.644 (19)°
*V* = 784.33 (15) Å^3^
*Z* = 2
Mo *K*α radiation
μ = 13.75 mm^−1^
*T* = 223 K
0.30 × 0.15 × 0.12 mm

#### Data collection

Rigaku Saturn diffractometer
Absorption correction: multi-scan (*REQAB*; Jacobson, 1998 ▶) *T*_min_ = 0.095, *T*_max_ = 0.191
6692 measured reflections
2912 independent reflections
2068 reflections with *I* > 2σ(*I*)
*R*_int_ = 0.075

#### Refinement

*R*[*F* ^2^ > 2σ(*F* ^2^)] = 0.045
*wR*(*F* ^2^) = 0.104
*S* = 1.08
2912 reflections
127 parameters
H-atom parameters constrained
Δρ_max_ = 1.74 e Å^−3^
Δρ_min_ = −2.02 e Å^−3^

### 

Data collection: *CrystalClear* (Rigaku, 2001 ▶); cell refinement: *CrystalClear*; data reduction: *CrystalStructure* (Rigaku, 2004 ▶); program(s) used to solve structure: *SHELXS97* (Sheldrick, 2008 ▶); program(s) used to refine structure: *SHELXL97* (Sheldrick, 2008 ▶); molecular graphics: *SHELXTL* (Sheldrick, 2008 ▶); software used to prepare material for publication: *SHELXTL*.

## Supplementary Material

Crystal structure: contains datablock(s) I, global. DOI: 10.1107/S1600536812031017/rz2782sup1.cif

Structure factors: contains datablock(s) I. DOI: 10.1107/S1600536812031017/rz2782Isup2.hkl

Additional supplementary materials:  crystallographic information; 3D view; checkCIF report

## Figures and Tables

**Fig. 1.**
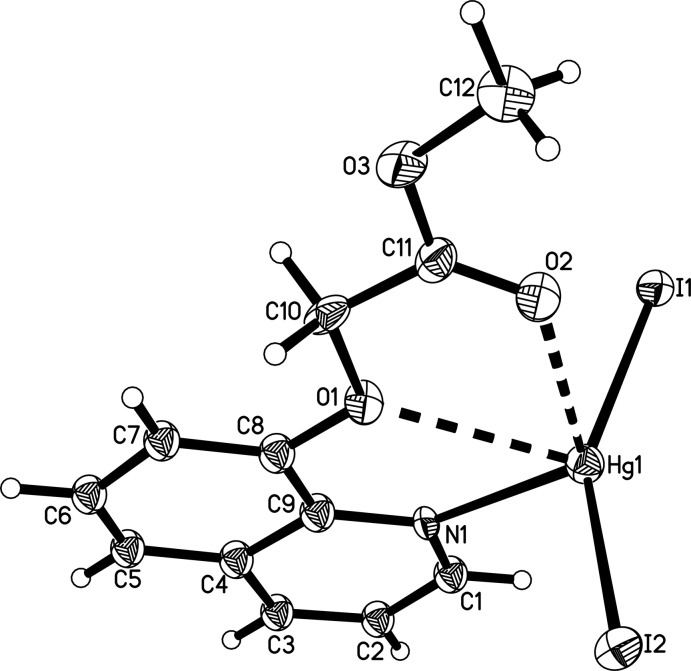
The molecular structure of the title compound with 30% probability displacement ellipsoids. The dashed lines indicate weak Hg···O interactions.

**Fig. 2.**
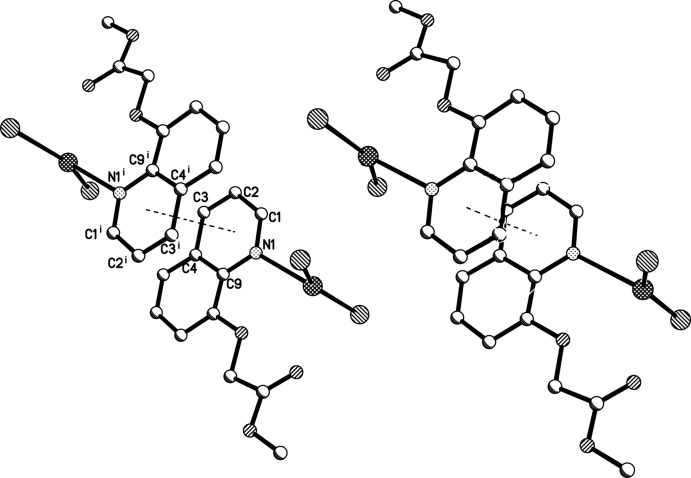
A view of intermolecular π-π stacking interactions between the quinoline rings of neighbouring complexes. H atoms are omitted for clarity. Symmetry code: (i) 1-x, 1-y, 1-z.

## supplementary crystallographic information

### Comment

In the past decades, the complexes of quinoline derivatives have been intensively studied due to their intriguing diversity and potential applications as functional materials (Cheng *et al.*, 2007; Ghedini *et al.*, 2002; Inomata *et al.*, 1999; Jotterand *et al.*, 2001). 8-Quinolinyloxyacetic acid and their derivatives exhibit a rich structural variety, and reports of the complexes with such ligands have increased in recent years (Cheng *et al.*, 2007; Song *et al.*, 2004; Wang, Song *et al.*, 2005; Wang, Fan *et al.*, 2008). As a contribution to this research field, we prepared the title Hg^II^ complex with 8-(methoxycarbonylmethoxy)quinoline ligand and report its crystal structure herein.

The title HgI_2_ adduct is a mononuclear compound. The Hg^II^ atom exists in a distorted trigonal planar geometry formed by two I atoms and one quinoline N atom of the 8-(methoxycarbonylmethoxy)quinoline ligand (Fig. 1). The Hg—N bond length is 2.470 (9) Å and the Hg—I bond lengths are 2.6163 (12) and 2.6246 (11) Å. The angles around the Hg atom vary from 102.2 (2) to 151.47 (4)°. Weak Hg···O interactions with distances of 2.764 (1) and 2.897 (1) Å are observed. Intermolecular face-to-face π-π stacking interactions are also observed between the quinoline rings of centrosymmetrically related complex molecules, with a centroid-centroid separation of 3.563 (9) Å (Fig. 2).

### Experimental

Triethylamine (0.0101 g, 0.1 mmol) and 8-quinolinyloxyacetic acid (0.0203 g, 0.1 mmol) were dissolved in methanol (3 ml). The mixture was stirred for 2 min, Then, the mixture and HgI_2_ (0.0455 g, 0.1 mmol) were placed in a thick Pyrex tube and heated at 150 °C for 5 days. After cooling at a rate of 5 °C/h to ambient temperature, colourless prism crystals were collected, washed with anhydrous ethanol, and dried at room temperature. The yield was 52% based on 8-quinolinyloxyacetic acid. Analysis found: C, 21.98; H, 1.63; N, 2.11%; calculated for C_12_H_11_I_2_HgNO_3_: C, 21.46; H, 1.65; N, 2.09%.

### Refinement

H atoms were included in calculated positions and refined as riding, with C—H distances of 0.94 (aromatic), 0.98 (methylene) and 0.97 Å (methyl), and with *U*_iso_(H) = 1.2*U*_eq_(C) or 1.5*U*_eq_(C) for methyl H atoms.

### Crystal data

**Table:**

[HgI_2_(C_12_H_11_NO_3_)]	*Z* = 2
*M**_r_* = 671.61	*F*(000) = 600
Triclinic, *P*1	*D*_x_ = 2.844 Mg m^−^^3^
Hall symbol: -P 1	Mo *K*α radiation, λ = 0.71075 Å
*a* = 7.5889 (5) Å	Cell parameters from 3945 reflections
*b* = 10.3670 (7) Å	θ = 3.0–27.5°
*c* = 11.4241 (11) Å	µ = 13.75 mm^−^^1^
α = 72.203 (18)°	*T* = 223 K
β = 74.40 (2)°	Block, colourless
γ = 68.644 (19)°	0.30 × 0.15 × 0.12 mm
*V* = 784.33 (15) Å^3^	

### Data collection

**Table:**

Rigaku Saturn diffractometer	2912 independent reflections
Radiation source: fine-focus sealed tube	2068 reflections with *I* > 2σ(*I*)
Graphite monochromator	*R*_int_ = 0.075
Detector resolution: 14.63 pixels mm^-1^	θ_max_ = 25.5°, θ_min_ = 3.0°
ω scans	*h* = −9→9
Absorption correction: multi-scan (*REQAB*; Jacobson, 1998)	*k* = −11→12
*T*_min_ = 0.095, *T*_max_ = 0.191	*l* = −13→11
6692 measured reflections	

### Refinement

**Table:**

Refinement on *F*^2^	Secondary atom site location: difference Fourier map
Least-squares matrix: full	Hydrogen site location: inferred from neighbouring sites
*R*[*F*^2^ > 2σ(*F*^2^)] = 0.045	H-atom parameters constrained
*wR*(*F*^2^) = 0.104	*w* = 1/[σ^2^(*F*_o_^2^) + (0.0218*P*)^2^] where *P* = (*F*_o_^2^ + 2*F*_c_^2^)/3
*S* = 1.08	(Δ/σ)_max_ < 0.001
2912 reflections	Δρ_max_ = 1.74 e Å^−^^3^
127 parameters	Δρ_min_ = −2.02 e Å^−^^3^
0 restraints	Extinction correction: *SHELXL97* (Sheldrick, 2008), Fc^*^=kFc[1+0.001xFc^2^λ^3^/sin(2θ)]^-1/4^
Primary atom site location: structure-invariant direct methods	Extinction coefficient: 0.0122 (6)

### Special details

**Table:**

Geometry. All e.s.d.'s (except the e.s.d. in the dihedral angle between two l.s. planes) are estimated using the full covariance matrix. The cell e.s.d.'s are taken into account individually in the estimation of e.s.d.'s in distances, angles and torsion angles; correlations between e.s.d.'s in cell parameters are only used when they are defined by crystal symmetry. An approximate (isotropic) treatment of cell e.s.d.'s is used for estimating e.s.d.'s involving l.s. planes.
Refinement. Refinement of *F*^2^ against ALL reflections. The weighted *R*-factor *wR* and goodness of fit *S* are based on *F*^2^, conventional *R*-factors *R* are based on *F*, with *F* set to zero for negative *F*^2^. The threshold expression of *F*^2^ > σ(*F*^2^) is used only for calculating *R*-factors(gt) *etc*. and is not relevant to the choice of reflections for refinement. *R*-factors based on *F*^2^ are statistically about twice as large as those based on *F*, and *R*- factors based on ALL data will be even larger.

### Fractional atomic coordinates and isotropic or equivalent isotropic displacement parameters (Å^2^)

**Table:**

	*x*	*y*	*z*	*U*_iso_*/*U*_eq_	
Hg1	0.51208 (7)	0.82132 (6)	0.69012 (5)	0.0363 (2)	
I1	0.20950 (11)	0.98926 (10)	0.59179 (9)	0.0367 (3)	
I2	0.79754 (12)	0.77753 (11)	0.80042 (9)	0.0407 (3)	
O1	0.4074 (12)	0.5817 (10)	0.8269 (8)	0.036 (2)	
O2	0.2395 (13)	0.8117 (11)	0.9230 (9)	0.044 (3)	
O3	0.0431 (12)	0.7026 (11)	1.0766 (9)	0.044 (3)	
N1	0.6241 (13)	0.6124 (11)	0.5978 (11)	0.032 (3)	
C1	0.7266 (17)	0.6246 (15)	0.4837 (13)	0.0351 (11)	
H1	0.7388	0.7148	0.4404	0.042*	
C2	0.8204 (17)	0.5104 (14)	0.4210 (13)	0.0351 (11)	
H2	0.8916	0.5259	0.3396	0.042*	
C3	0.8046 (17)	0.3814 (15)	0.4808 (13)	0.0351 (11)	
H3	0.8640	0.3053	0.4409	0.042*	
C4	0.6940 (18)	0.3573 (15)	0.6095 (13)	0.0351 (11)	
C5	0.6720 (17)	0.2266 (15)	0.6712 (13)	0.0351 (11)	
H5	0.7304	0.1489	0.6334	0.042*	
C6	0.5686 (17)	0.2116 (15)	0.7835 (13)	0.0351 (11)	
H6	0.5565	0.1212	0.8261	0.042*	
C7	0.4726 (17)	0.3280 (14)	0.8446 (13)	0.0351 (11)	
H7	0.3990	0.3159	0.9255	0.042*	
C8	0.4950 (18)	0.4584 (15)	0.7775 (13)	0.0351 (11)	
C9	0.6036 (18)	0.4816 (15)	0.6632 (13)	0.0351 (11)	
C10	0.2660 (17)	0.5716 (16)	0.9448 (14)	0.041 (4)	
H10A	0.1636	0.5416	0.9346	0.050*	
H10B	0.3295	0.5001	1.0119	0.050*	
C11	0.1829 (19)	0.7104 (17)	0.9780 (15)	0.040 (4)	
C12	−0.052 (2)	0.8306 (17)	1.1242 (14)	0.056 (5)	
H12A	0.0373	0.8482	1.1598	0.083*	
H12B	−0.1621	0.8181	1.1881	0.083*	
H12C	−0.0946	0.9109	1.0565	0.083*	

### Atomic displacement parameters (Å^2^)

**Table:**

	*U*^11^	*U*^22^	*U*^33^	*U*^12^	*U*^13^	*U*^23^
Hg1	0.0374 (3)	0.0355 (4)	0.0358 (4)	−0.0110 (2)	−0.0080 (2)	−0.0067 (3)
I1	0.0336 (5)	0.0352 (6)	0.0399 (6)	−0.0105 (4)	−0.0073 (4)	−0.0065 (5)
I2	0.0408 (5)	0.0497 (7)	0.0322 (6)	−0.0198 (4)	−0.0088 (4)	−0.0011 (5)
O1	0.045 (5)	0.034 (6)	0.026 (6)	−0.013 (4)	0.013 (4)	−0.018 (5)
O2	0.052 (6)	0.045 (7)	0.036 (7)	−0.019 (5)	0.012 (5)	−0.019 (6)
O3	0.046 (5)	0.055 (7)	0.028 (6)	−0.022 (5)	0.017 (4)	−0.017 (6)
N1	0.030 (6)	0.018 (6)	0.050 (8)	−0.005 (5)	−0.011 (5)	−0.010 (6)
C1	0.038 (2)	0.032 (3)	0.032 (3)	−0.011 (2)	−0.0028 (19)	−0.005 (2)
C2	0.038 (2)	0.032 (3)	0.032 (3)	−0.011 (2)	−0.0028 (19)	−0.005 (2)
C3	0.038 (2)	0.032 (3)	0.032 (3)	−0.011 (2)	−0.0028 (19)	−0.005 (2)
C4	0.038 (2)	0.032 (3)	0.032 (3)	−0.011 (2)	−0.0028 (19)	−0.005 (2)
C5	0.038 (2)	0.032 (3)	0.032 (3)	−0.011 (2)	−0.0028 (19)	−0.005 (2)
C6	0.038 (2)	0.032 (3)	0.032 (3)	−0.011 (2)	−0.0028 (19)	−0.005 (2)
C7	0.038 (2)	0.032 (3)	0.032 (3)	−0.011 (2)	−0.0028 (19)	−0.005 (2)
C8	0.038 (2)	0.032 (3)	0.032 (3)	−0.011 (2)	−0.0028 (19)	−0.005 (2)
C9	0.038 (2)	0.032 (3)	0.032 (3)	−0.011 (2)	−0.0028 (19)	−0.005 (2)
C10	0.033 (7)	0.053 (10)	0.033 (9)	−0.024 (7)	−0.010 (6)	0.014 (8)
C11	0.041 (8)	0.038 (9)	0.040 (10)	−0.018 (7)	−0.016 (7)	0.007 (8)
C12	0.068 (10)	0.064 (12)	0.029 (10)	−0.024 (9)	0.029 (8)	−0.030 (9)

### Geometric parameters (Å, º)

**Table:**

Hg1—N1	2.470 (9)	C4—C5	1.371 (19)
Hg1—I1	2.6163 (12)	C4—C9	1.456 (17)
Hg1—I2	2.6246 (11)	C5—C6	1.308 (18)
O1—C8	1.419 (14)	C5—H5	0.9400
O1—C10	1.482 (15)	C6—C7	1.441 (16)
O2—C11	1.209 (17)	C6—H6	0.9400
O3—C11	1.328 (17)	C7—C8	1.381 (19)
O3—C12	1.452 (15)	C7—H7	0.9400
N1—C1	1.321 (17)	C8—C9	1.348 (19)
N1—C9	1.377 (17)	C10—C11	1.469 (19)
C1—C2	1.432 (16)	C10—H10A	0.9800
C1—H1	0.9400	C10—H10B	0.9800
C2—C3	1.336 (19)	C12—H12A	0.9700
C2—H2	0.9400	C12—H12B	0.9700
C3—C4	1.481 (19)	C12—H12C	0.9700
C3—H3	0.9400		
			
N1—Hg1—I1	104.4 (2)	C7—C6—H6	118.7
N1—Hg1—I2	102.2 (2)	C8—C7—C6	116.1 (13)
I1—Hg1—I2	151.47 (4)	C8—C7—H7	121.9
C8—O1—C10	117.4 (10)	C6—C7—H7	121.9
C11—O3—C12	116.1 (11)	C9—C8—C7	124.7 (12)
C1—N1—C9	118.9 (10)	C9—C8—O1	113.5 (12)
C1—N1—Hg1	117.2 (9)	C7—C8—O1	121.7 (12)
C9—N1—Hg1	123.5 (9)	C8—C9—N1	123.9 (12)
N1—C1—C2	124.8 (13)	C8—C9—C4	114.8 (13)
N1—C1—H1	117.6	N1—C9—C4	121.2 (12)
C2—C1—H1	117.6	C11—C10—O1	109.9 (12)
C3—C2—C1	118.5 (13)	C11—C10—H10A	109.7
C3—C2—H2	120.7	O1—C10—H10A	109.7
C1—C2—H2	120.7	C11—C10—H10B	109.7
C2—C3—C4	120.5 (12)	O1—C10—H10B	109.7
C2—C3—H3	119.8	H10A—C10—H10B	108.2
C4—C3—H3	119.8	O2—C11—O3	127.6 (13)
C5—C4—C9	122.5 (13)	O2—C11—C10	123.5 (13)
C5—C4—C3	121.4 (11)	O3—C11—C10	108.8 (13)
C9—C4—C3	116.1 (12)	O3—C12—H12A	109.5
C6—C5—C4	119.1 (12)	O3—C12—H12B	109.5
C6—C5—H5	120.4	H12A—C12—H12B	109.5
C4—C5—H5	120.4	O3—C12—H12C	109.5
C5—C6—C7	122.6 (14)	H12A—C12—H12C	109.5
C5—C6—H6	118.7	H12B—C12—H12C	109.5
			
I1—Hg1—N1—C1	80.1 (9)	C7—C8—C9—N1	−180.0 (12)
I2—Hg1—N1—C1	−89.5 (9)	O1—C8—C9—N1	−1.9 (19)
I1—Hg1—N1—C9	−107.3 (9)	C7—C8—C9—C4	2 (2)
I2—Hg1—N1—C9	83.1 (9)	O1—C8—C9—C4	−179.4 (10)
C9—N1—C1—C2	−0.1 (19)	C1—N1—C9—C8	−176.7 (13)
Hg1—N1—C1—C2	172.9 (9)	Hg1—N1—C9—C8	10.8 (18)
N1—C1—C2—C3	0 (2)	C1—N1—C9—C4	0.7 (18)
C1—C2—C3—C4	−0.7 (19)	Hg1—N1—C9—C4	−171.9 (8)
C2—C3—C4—C5	178.3 (13)	C5—C4—C9—C8	−0.7 (19)
C2—C3—C4—C9	1.2 (18)	C3—C4—C9—C8	176.4 (12)
C9—C4—C5—C6	−1 (2)	C5—C4—C9—N1	−178.3 (12)
C3—C4—C5—C6	−178.1 (12)	C3—C4—C9—N1	−1.2 (18)
C4—C5—C6—C7	1 (2)	C8—O1—C10—C11	−177.0 (10)
C5—C6—C7—C8	0.2 (19)	C12—O3—C11—O2	0 (2)
C6—C7—C8—C9	−2 (2)	C12—O3—C11—C10	178.5 (11)
C6—C7—C8—O1	179.8 (10)	O1—C10—C11—O2	−7.3 (19)
C10—O1—C8—C9	172.5 (11)	O1—C10—C11—O3	173.7 (10)
C10—O1—C8—C7	−9.4 (17)		
